# Inspired by Skeletal Muscles: Study of the Physical and Electrochemical Properties of Derived Lignocellulose-Based Carbon Fibers

**DOI:** 10.3390/ma15228068

**Published:** 2022-11-15

**Authors:** Xing Gao, Ying Zhang, Yueting Wu, Tat Thang Nguyen, Jie Wu, Minghui Guo, Chunhua Du

**Affiliations:** 1 College of Sports and Human Sciences, Post-Doctoral Mobile Research Station, Graduate School, Harbin Sport University, Harbin 150008, China; 2 Key Laboratory of Bio-Based Material Science and Technology (Ministry of Education), College of Material Science and Engineering, Northeast Forestry University, Harbin 150040, China; 3 College of Wood Industry and Interior Design, Vietnam National University of Forestry, Hanoi 156220, Vietnam

**Keywords:** lignocellulose, chemical crosslinking, carbon fiber, electrochemical properties

## Abstract

Skeletal muscles exhibit excellent properties due to their well-developed microstructures. Taking inspiration from nature that thick filaments and thin filaments are linked by “cross-bridges”, leading to good stability and ion transport performance of muscles. In this work, extracted poplar lignin and microcrystalline cellulose (MCC) were connected by biomimetic covalent bonds, akin to biological muscle tissue, in which isophorone diisocyanate was used as the chemical crosslinking agent. Then, poplar lignin–MCC was mixed with polyacrylonitrile to serve as the precursor for electrospinning. The results show that due to the effective covalent-bond connection, the precursor fibers possess excellent morphology, smooth surface, good thermal stability, and high flexibility and toughness (average elongation-at-break is 51.84%). Therefore, after thermal stabilization and carbonization, derived lignocellulose-based carbon fibers (CFs) with a reduced cost, complete fiber morphology with a uniform diameter (0.48 ± 0.22 μm), and high graphitization degree were obtained. Finally, the electrodes fabrication and electrochemical testing were carried out. The results of electrochemical impedance spectroscopy (EIS) indicate that the Rs and Rct values of CFs supercapacitors are 1.18 Ω and 0.14 Ω, respectively. Results of cyclic voltammetry (CV) and galvanostatic charge–discharge (GCD) suggest that these CFs demonstrate great application potential in electrochemical materials.

## 1. Introduction

Carbon fibers (CFs), known as the “king of new materials”, are special fibers with a carbon content of greater than 90%. Owing to their excellent properties, such as lightweight nature, high strength, good corrosion resistance, and high temperature resistance, CFs have been widely used in aerospace and automobile industries, biomedicine, sports, energy generation, and other industries [1,2,3]. The industrial processing steps of CFs include spinning, stabilization, carbonization, and graphitization [4]. Electrospinning is a cost-effective and simple technology to realize the industrial production of sub-micro precursor fibers (PFs), which can be used to easily prepare continuous CFs [5]. Commercially, sub-micro PFs exhibit unique properties, demonstrating application prospects for batteries, environmental remediation, electrical and optical sensors, and the textile industry [6,7]. Therefore, the processing scale of sub-micrometer-diameter PFs has been gradually expanded in the global fiber market [8,9]. Currently, commercial fibers are produced almost entirely from non-renewable petroleum-based materials, which are expensive and not environmentally friendly [5,10,11,12]. Therefore, reducing fiber costs and exploring renewable precursors have become the focus of the research and development of fiber products.

Lignocellulosic biomass is an ideal candidate for the production of fiber products due to its high carbon content, high aromaticity, renewability, environmentally friendly characteristics, and cost-effectiveness [5,13,14,15]. Among the types of biomasses, lignin is an amorphous polymer with poor mechanical properties, making it difficult to withstand high drafting tension during spinning [16]. Cellulose exhibits good flexibility; however, it is difficult to obtain CFs by carbonization due to the low heat resistance [10]. Therefore, blending the two materials to form composite fibers via the interaction between their polar functional groups can not only overcome their disadvantages but also integrate the superior thermodynamic stability of lignin with the good spinnability of cellulose [17,18,19,20]. However, owing to differences in the physicochemical properties (such as solubility, plasticity, and oxygen content) of lignin and cellulose, their physical blending is limited by weak intermolecular interactions, leading to an apparent phase separation during thermal stabilization [21,22]. Morphological collapse is a major challenge that limits the development of lignocellulose biomass-derived fibers [5]. Related research is still in the nascent stage.

Meanwhile, soft tissues of organisms exhibit unique properties due to their well-developed microstructures [23]. Skeletal muscle tissues comprise numerous highly oriented, tightly packed muscle fiber bundles with transport channels for ions and small molecules. They are essential for biological functions of muscles, including highly anisotropic mechanical properties, contraction of muscles and signal transport of neurons [24,25,26,27]. Myofibrils in the muscle fibers comprise a thick filament and a thin filament, which are packed in a regular striated arrangement. The heads of myosins on the thick filaments protrude outward to form a “cross-bridge”, which can reversibly bind to actin on the thin filaments under certain conditions; such reversible binding leads to the “sliding filament” process and subsequently results in muscle contraction [28]. Such tissue properties of myofibrils (thick filaments and thin filaments are linked by “cross-bridges”) exhibit improved properties which are reflected in both their stable structure and ion transport. Therefore, introducing the characteristic connections between filaments into lignin-celluloses blends via a chemical crosslinking would be an effective and interesting biomimetic synthesis method to obtain fiber products with excellent morphology and properties. Therefore, this study developed lignocellulose-based CFs with good physical and electrochemical properties by linking the lignin extracted from poplar wood with MCC via biomimetic covalent bonds, which are formed by the urethane reaction between isocyanate groups in isophorone diisocyanate and hydroxyl groups. The nature-inspired design of biomimetic muscles can integrate the advantages of biological tissues such as flexibility, electrochemical characteristics, mechanical properties, and physical and chemical properties, representing a new direction for the design of advanced multifunctional materials in the future.

## 2. Experimental

### 2.1. Materials and Reagents

Poplar wood powder (100 mesh) was purchased from Shijiazhuang Yuxin Building Materials Company of China. Reagents including ethyl alcohol, hydrochloric acid, and sodium hydroxide were purchased from Tianjin Dongda Chemical Reagent Factory of China. Microcrystalline cellulose (MCC), isophorone diisocyanate (IPDI), dibutyltin dilaurate, N,N-dimethylformamide (DMF), and polyacrylonitrile (PAN) were purchased from Shanghai Dingfen Chemical Technology Company of China. All chemical reagents were used without further purification. Deionized water was used in all synthetic procedures.

### 2.2. Extraction of Lignin

The experimental methods about extraction of lignin were carried out by referring to reference [29]. Poplar wood powder (50 g) was immersed into a 5% sodium hydroxide solution (500 mL) and stirred at 80 °C for 4 h. The reacted mixture was centrifuged to obtain a liquid supernatant, and its pH was adjusted to 2 using 37% hydrochloric acid, followed by standing for 12 h. Next, the reacted mixture was centrifuged, and the obtained precipitate was washed repeatedly and dried at 60 °C for 48 h to afford a brown solid. After the solid was ground, the powder was denoted as “poplar lignin”, which was used for subsequent characterization and synthesis.

### 2.3. Preparation of Lignocellulose-Based Precursor

First, 0.5 g of poplar lignin and 0.5 g of MCC were dissolved in 18 g of DMF solvent and stirred at 60 °C for 2 h in a water bath. Then, IPDI (2 wt%) and dibutyltin dilaurate (catalyst, 0.1 wt%) were added, and the mixture was maintained for 12 h. Subsequently, 2 g of PAN was added and stirred at normal temperatures for 6 h. Finally, a uniform and viscous brown blended spinning solution without precipitates was formed and used as the lignocellulose-based precursor of CFs, which was denoted as “L-precursor” for subsequent characterization.

### 2.4. Preparation of Carbon Fiber

PFs were obtained by electrospinning. First, 5 mL of the blended spinning solution was added into the syringe, which was fixed to a syringe pump, and the PFs were collected using a rotating drum, along with cooperation by the reciprocating platform to improve the uniformity and receiving area of the PFs. The following electrospinning conditions were set: a feed rate of 16.7 uL/min, an applied voltage/power of 24 kV, a receiving distance of 15 cm, a drum speed of 700 rpm, a reciprocating motion of 340 r, an environment temperature of 31 °C, and a humidity of 70 %rh. After spinning for 7 h, the light gray PF membrane with a size of an A4 paper was collected and dried at 60 °C.

**Scheme 1:**
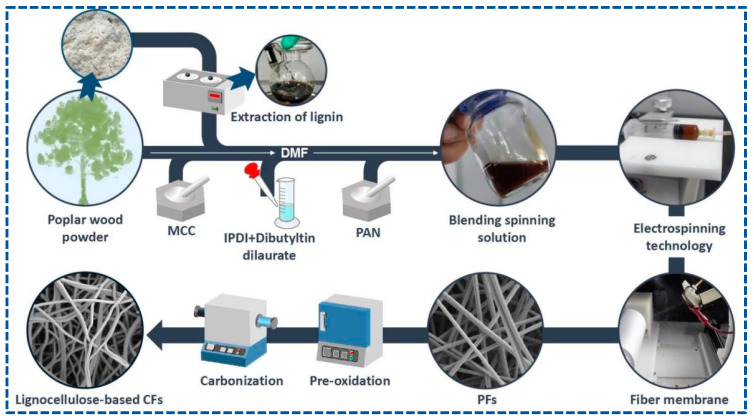
Concise schematic of the synthetic process of lignocellulose-based CFs.

Pre-oxidation of PFs was performed in a muffle furnace. PFs were heated at 220 °C under air for 12 h to obtain stabilized fibers (SFs). The carbonization of SFs was conducted in a tubular furnace at a heating temperature of 1000 °C for 2 h under nitrogen. Finally, lignocellulose-based CFs were obtained. Scheme 1 shows the concise synthesis.

### 2.5. Supercapacitor Device Fabrication

The two-electrode symmetric supercapacitor device was described as follows: A uniform slurry containing 80 wt% CFs, 10 wt% polytetrafluoroethylene (PTFE), and 10 wt% carbon black dispersed in a DMF solution was pasted onto nickel foam and dried at 80 °C for 12 h under vacuum. Then, two working electrodes were separated by a polypropylene film, and KOH was used as the electrolyte.

### 2.6. Characterization

Sample morphologies were observed by scanning electron microscopy (SEM, Zeiss Gemini 300, Jena, Germany). Quantitative ^13^C NMR spectra of samples were recorded on a Bruker Avance 400 MHz spectrometer, and NMR data were analyzed using Topspin 2.1 software (Bruker, Germany). Fourier transform infrared (FTIR) spectra were recorded on an FTIR spectrometer (Nicolet iS 10, Shanghai, China) under an operating frequency of 450–4000 cm^−1^. X-ray diffraction (XRD) patterns were recorded on an X-ray diffractometer (D8-Advance, Bruker, Germany) at a scanning speed of 10°/min. X-ray photoelectron spectra (XPS) were recorded on an X-ray photoelectron spectrometer (Thermo Kalpha, Shenzhen, China). Raman spectra were recorded on a laser Raman spectrometer (LabRam HR Evolution, Shenzhen, China) at an excitation wavelength of 532 nm. Differential scanning calorimetry (DSC) analysis and thermogravimetry analysis (TGA) were conducted on an SDT Q500 system (TA Instruments, Shanghai, China). The sample was heated at a heating rate of 10 °C/min from room temperature to 700 °C under nitrogen. Nitrogen adsorption–desorption isotherms of the samples were measured using an automated surface and pore size analyzer (Mike ASAP2460, Norcross, GA, USA). The contact angle between the surface of the sample and water drop was measured using a contact angle measuring instrument (Dataphysics OCA20, Filderstadt, Germany). The elongation-at-break test was conducted on an electronic universal testing machine (CMT6103, Eden Prairie, MN, USA). The length and width of the specimens were 40 mm and 10 mm, respectively. The electrochemical properties of the supercapacitor device were investigated by electrochemical impedance spectroscopy (EIS), cyclic voltammetry (CV), and galvanostatic charge–discharge (GCD) test on an electrochemical workstation (CHI 760E, Shanghai, China). KOH was used as the electrolyte.

## 3. Results and Discussion

### 3.1. Properties of Poplar Lignin

**Figure 1:**
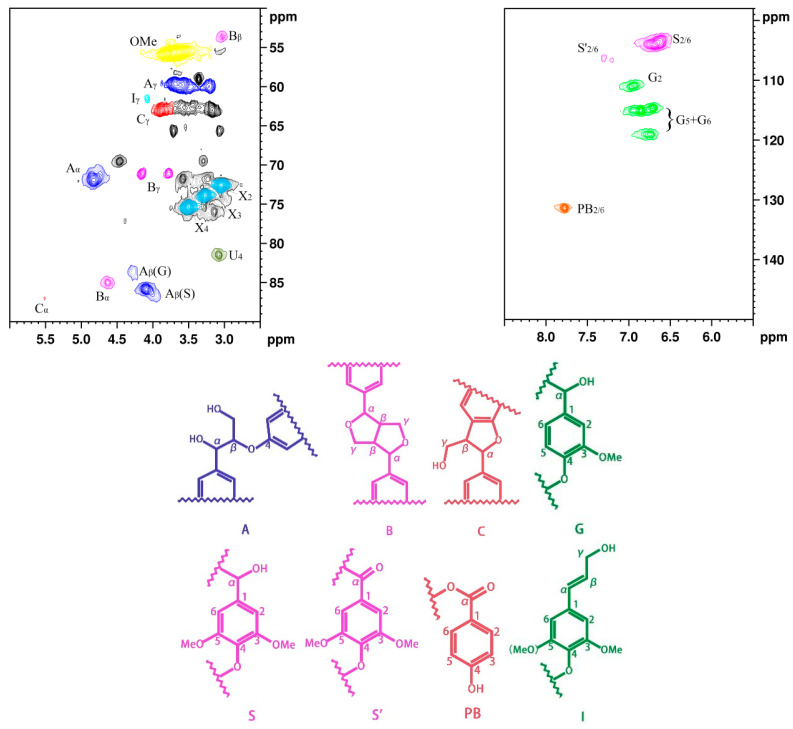
NMR spectrum of poplar lignin and the main chemical structures and linking modes.

Figure 1 shows NMR spectrum (2D-HSQC), main chemical structures, and linkages of poplar lignin. The carbon atoms on the benzene ring are numbered from 1 to 6, and the carbon atoms of the benzene-ring side chains are named *α*, *β*, and *γ* [29]. The NMR spectrum can be divided into the aliphatic side-chain and aromatic-chain regions [5,30,31]. The aliphatic side-chain region (*δ_C_*/*δ_H_* 50–90/2.5–6.0 ppm) provides bond connections between units in lignin, such as the methoxy (*δ_C_*/*δ_H_* 56.2/3.69 ppm) and aryl ether bond *β*–O–4 (*δ_C_*/*δ_H_* 84.1/4.24 ppm) groups, while the main cross signals in the aromatic-chain region (*δ_C_*/*δ_H_* 100–150/5.5–8.5 ppm) correspond to the aromatic rings of the syringyl (S) and guaiacol (G) units in lignin, as well as some lignin substructures [32]. The results indicate that the main linkages between the monomeric structures are *β*–O–4, *β*–*β*, and *β*–5 bonds. In the aliphatic side-chain region (*δ_C_*/*δ_H_* 50–90/2.5–6.0 ppm), the *β*–O–4 linkage bonds are mainly associated with structure A, where the signal peak positions of A*α*, A*β*, and A*γ* are observed at *δ_C_*/*δ_H_* 72.43/4.79, 86.4/4.07, and 60.3/3.59 ppm, respectively. The signal peak positions of B*α*, B*β*, and B*γ* in structure B are observed at *δ_C_*/*δ_H_* 85.7/4.60, 54.3/3.01, and 3.75/71.8 ppm, respectively. In the aromatic-chain region (*δ_C_*/*δ_H_* 100–150/5.5–8.5 ppm), the signal peaks for CH-2/6 in the S monomer are observed at *δ_C_*/*δ_H_* 104.5/6.72 ppm, and CH-2, 5, and 6 in the G monomer are located at *δ_C_*/*δ_H_* 111.5/6.89, 115.6/6.68, and 119.5/5.71 ppm, respectively. These lignin constituent abundances and S/G values were calculated by quantitative 2D-HSQC NMR spectrum (Supplementary Information). The contents of *β*–O–4, *β*–*β*, and *β*–5 are observed at 56.57, 6.45, and 2.07, respectively, and the S/G ratio is 1.54 (Table S1). The results indicated that poplar lignin exhibits relatively abundant *β*–O–4 bonds; thus, poplar lignin exhibits linear structures [33,34,35].

The thermal stability of lignin exerts a key effect on the morphology of the produced PFs [5,33]. Figure S1 in Supporting Information shows the TG and DTG curves of poplar lignin. As can be observed, the thermal decomposition process is divided into three main stages: the first weight loss stage occurs at ~76.7 °C, corresponding to the volatilization of the water present in the sample; the second weight loss stage occurs at ~241.3 °C, corresponding to the dissociation of the *β*–O–4 bond, the removal of the side chain of lignin, and the weight loss of residual hemicellulose [29]; the third weight loss stage occurs at ~337.5 °C, corresponding to the decomposition of the lignin units [36]. After thermal decomposition, the final residual quality of the sample is 35.31%. This result reveals that poplar lignin exhibits good thermal stability.

### 3.2. Properties of Lignocellulose-Based Precursor

**Scheme 2:**
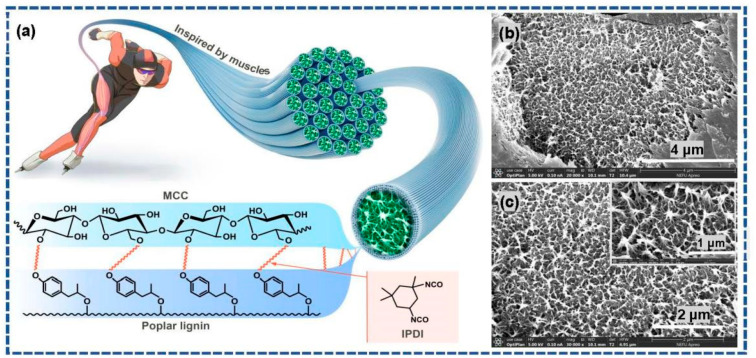
(**a**) Schematic and chemical crosslinking modification mechanism of biomimetic skeletal muscle tissues; (**b**,**c**) SEM images of the L-precursor.

The hydroxyl groups in poplar lignin and MCC serve as the active reaction sites. In addition, IPDI contains two isocyanate groups, which can be used as chemical crosslinking agents. Therefore, based on the principles of muscle contraction, poplar lignin and MCC imitate the molecular structure characteristics in muscles: myosin on the thick filament is bound to actin on the thin filament via a cross-bridge. The urethane reaction occurs easily between the isocyanate groups and hydroxyl groups via chemical crosslinking modification to form a stable covalent linkage [5]. Scheme 2b,c show the morphologies of the L-precursor. As can be observed in the exposed parts of the SEM image as well as its magnified image (inset in Scheme 2c), the three-dimensional porous network structure, closely resembling the microstructures of the myofibril cross-section of striated skeletal muscle tissues, is clearly observed.

**Figure 2:**
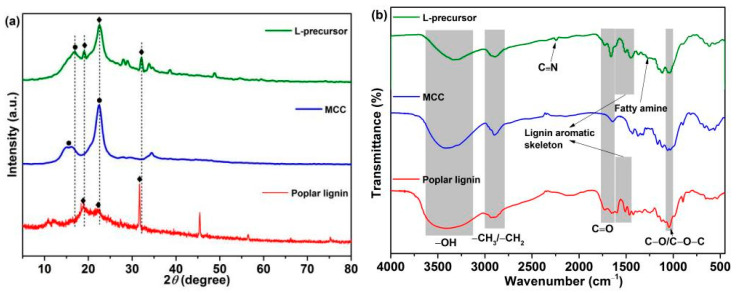
(**a**) XRD and (**b**) FTIR spectra of the samples.

Figure 2 shows the XRD (Figure 2a) and FTIR (Figure 2b) spectral analyses of the sample structures. Diffraction peaks are observed at 2*θ* = ~15.4° and 22.5° in the XRD pattern of MCC, corresponding to the type Ⅰ crystal lattice of cellulose [37,38,39]. The typical diffraction peaks centered at 2*θ* = ~18.7°, 22.4°, and 31.7° are observed in the XRD pattern of poplar lignin, possibly corresponding to the alkyl, hydroxyl, and carbonyl groups in the lignin structure [40,41]. Typical diffraction peaks corresponding to MCC and poplar lignin are observed at 2*θ* = ~16.9°, 19.0°, 22.6°, and 32.1° in the XRD pattern of the L-precursor, which shift slightly to a higher angle than those in the pure samples. This phenomenon indicates that chemical bonds between poplar lignin and MCC may exist. Figure 2b shows FTIR spectra of samples. The –OH stretching vibrations are observed between 3100 and 3500 cm^−1^; the bands between 2700 and 3000 cm^−1^ are attributed to the C–H stretching vibrations of the alkyl group [37,38,39]; the characteristic absorption bands are observed at 1645, 1647, 1660, 1730, and 1733 cm^−1^, corresponding to C=O derived from aldehyde and carboxyl [42]; and the bands at 1040, 1050, and 1052 cm^–1^ correspond to C–O/C–O–C stretching vibrations. The IR absorption peaks of poplar lignin at 1601, 1513, and 1464 cm^−1^ are attributed to characteristic stretching vibrations of the aromatic skeleton [29,43], which are observed in the FTIR spectrum of the L-precursor at 1610, 1510, and 1450 cm^−1^, respectively. Notably, compared with those of poplar lignin and MCC, the peak intensity of the –OH band in the FTIR spectrum of L-precursor decreases, and a new IR peak is observed at 1261 cm^−1^, corresponding to fatty amine [5,44,45]. This phenomenon is caused by the occurrence of the urethane reaction and consumption of the partial hydroxyl groups. In addition, the characteristic absorption of C≡N derived from PAN in the L-precursor is observed at 2240 cm^−1^.

**Figure 3:**
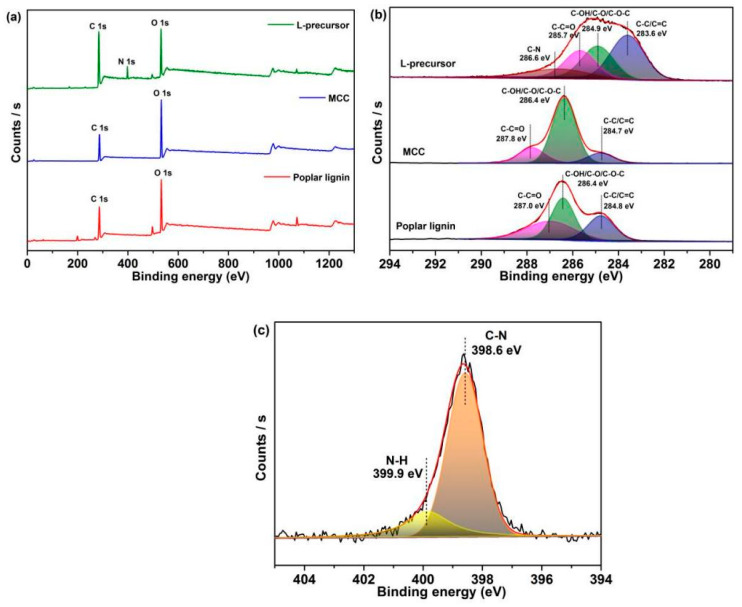
(**a**) XPS survey spectra of the samples. High-resolution XPS (**b**) C 1s spectrum and (**c**) N 1s spectrum of the samples.

XPS (Figure 3) shows characterization data related to the surface chemical structures of samples. Only C 1s and O 1s peaks are observed in the XPS spectrum of poplar lignin and MCC, while in the XPS spectrum of the L-precursor, except for C 1s (285.1 eV) and O 1s (532.4 eV) peaks, the N 1s peak (398.0 eV) is observed due to the introduction of nitrogen-containing functional groups (Figure 3a). Table S2 shows the elemental compositions of the samples. A small amount of stannum (Sn) derived from dibutyltin dilaurate is detected in the XPS spectrum of the L-precursor. The C 1s spectrum of poplar lignin can be fitted to three peaks at 284.8, 286.4, and 287.8 eV, respectively, and that of MCC reveals three peaks at 284.7, 286.4, and 287.8 eV, both corresponding to C–C/C=C, C–OH/C–O/C–O–C, and C–C=O, respectively (Figure 3b) [46]. Notably, a new fitting peak is observed at 286.6 eV for the L-precursor, which is attributed to C–N in the fatty amine, which is consistent with FTIR results [47,48]. Furthermore, C–C/C=C, C–OH/C–O/C–O–C, C–C=O (283.6, 284.9, and 285.9 eV, respectively) in the L-precursor are shifted to binding energies less than those of poplar lignin and MCC. A strong interaction in the L-precursor is speculated. The N 1s spectrum of the L-precursor (Figure 3c) is deconvoluted into two peaks at 398.6 and 399.9 eV, which are attributed to C–N and N–H in the fatty amine, respectively [46]. Based on the above results, the urethane reaction occurs, and covalent bonds are formed in the L-precursor.

### 3.3. Properties of Precursor Fibers

**Figure 4:**
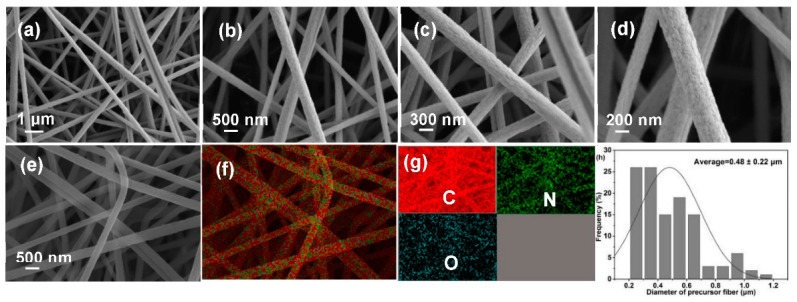
(**a**–**d**) SEM images, (**e**–**g**) EDX mapping, and (**h**) diameter distribution diagram of PFs.

Morphological collapse is a major challenge for lignocellulose-based PFs, which is closely related to precursor characteristics. SEM images (Figure 4a–d), EDX mapping images (Figure 4e–g), and diameter distribution diagram (Figure 4h) were recorded to analyze morphologies of PFs. Clearly, PFs exhibit a complete fiber morphology with a uniform diameter (0.48 ± 0.22 μm) and smooth surface. In addition, EDX images reveal the uniform distribution of C, O, and N in PFs, and the percentages of various elements are presented in Figure S2. These results preliminarily indicate that the excellent morphology possibly exerts a positive effect on the properties of the produced CFs.

**Figure 5:**
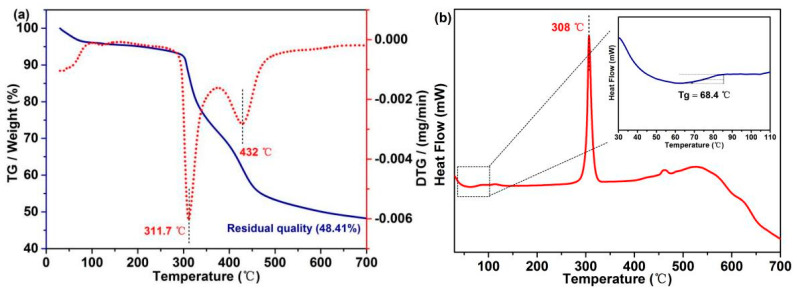
(**a**) TG and DTG curves and (**b**) DSC curves of PFs.

Excellent thermodynamic stability of PFs can effectively maintain the weight and filiform morphology of CFs. TG and DTG curves (Figure 5a) indicate that a thermal platform at ~100 °C is mainly attributed to water evaporation. With the increase in the temperature, the weight of PFs starts to decrease at 295.3 °C due to the decomposition of *β*–O–4, and the maximum decomposition rate is observed at ~311.7 °C. In addition, the degradation of C–C bonds starts from 392 °C, and the maximum decomposition rate is observed at ~432 °C. The final yield of PFs is 48.41%, indicating that the PFs exhibit a good stability and high yield. The phase transition behavior of PFs was determined by DSC, which is a key thermodynamic property [5]. The DSC curve of PFs exhibits a sharp peak pattern at ~308 °C, corresponding to the melting point of PAN (Figure 5b). To reveal additional details, the 30–110 °C region in the DSC curve was magnified (inset in Figure 5b), and PFs exhibit a single glass transition region (Tg = 68.4 °C), indicating that poplar lignin and MCC form a macromolecule via biomimetic covalent-bond linkages.

**Figure 6:**
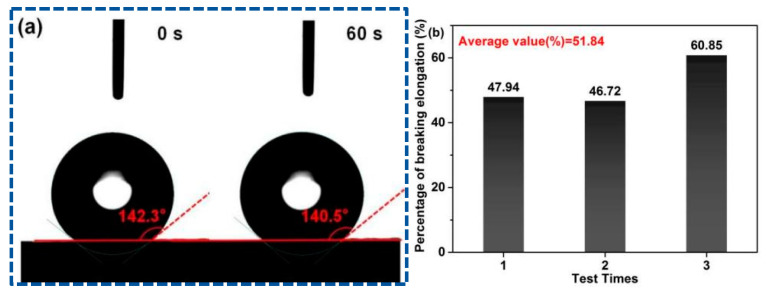
(**a**) Contact angles at 0 s and 60 s, and (**b**) percentage of breaking elongation of PFs.

Contact angle measurements were employed to observe the water wetting behavior of PFs. The PAN fiber surface exhibits strong hydrophobicity due to the absence of oxygen-containing functional groups in PAN, such as hydroxyl and carboxyl groups. Its water wetting behavior is described as follows: Droplets cannot fall on the surface [49]. The contact angle of the droplet on the PF membrane surface is 140.5° at 60 s (Figure 6a). This phenomenon is attributed to the introduction of hydrophilic groups via poplar lignin and MCC in the L-precursor. The enhanced hydrophilic property can reduce the phase separation and double the diffusion speed during solidification and reduce the formation of defects in PFs. Therefore, it is conducive to control the PF morphology.

Elongation-at-break is utilized for the characterization of the flexibility and toughness of fibers, which is one of the key indices that determine the service performance of fiber products. Fibers with a high elongation-at-break feel soft, and these fibers buffer the force received during processing in the textile industry; thus, the final products are not deformed easily. Figure 6b shows the elongation-at-break of the PF membrane, and the average elongation-at-break is calculated as 51.84% by triplicate tests, which is considerably greater than the normal standard requirements (15–30%). In addition to the improved hydrophilicity, the covalent-bond linkage between poplar lignin and MCC may be another important reason for the high flexibility and toughness of PFs.

### 3.4. Properties of Carbon Fibers

**Figure 7:**
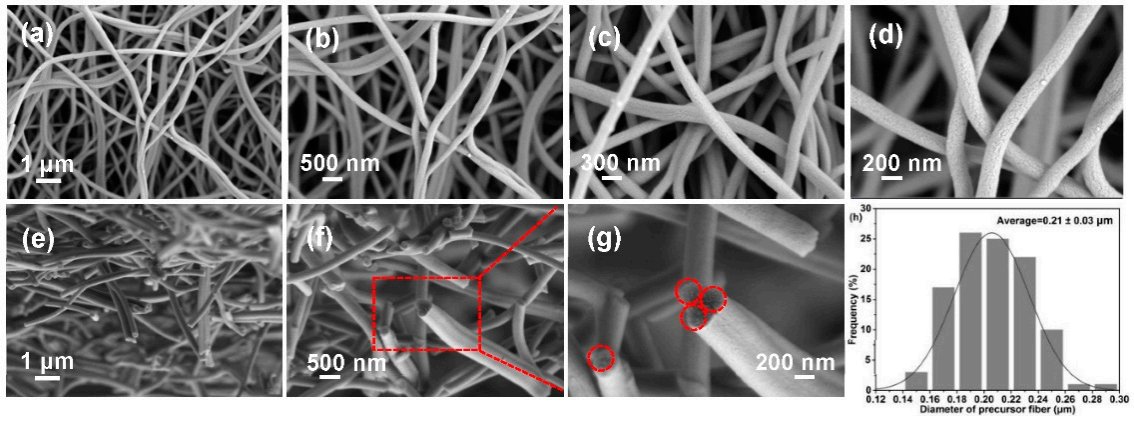
SEM images of (**a**–**d**) surfaces and (**e**–**g**) cross-sections of CFs, and (**h**) their diameter dis-tribution diagram.

Micro-morphology significantly affects CF performance. The average diameter of the produced CFs is 0.21 ± 0.03 μm (Figure 7h), which decrease significantly in comparison with those of PFs due to the removal of other impurities during carbonization. Clearly, the CFs maintain a complete and independent filiform morphology with a uniform diameter and no clear defects (Figure 7a–d). To further analyze the morphology, SEM images of cross-sections of CFs were recorded, as shown in Figure 7e–g. CFs exhibit relatively regular and neat cylindrical cross-sections (the dotted circles). The introduction of a biomimetic covalent bond between poplar lignin and MCC may be the origin of this excellent property, and the derived PFs exhibit an excellent morphology and thermal stability; hence, CFs with a satisfactory morphology are produced. This CF structure provides an internetworked pathway for promoting the rapid migration of electrolyte ions, which can benefit the electrochemical performance of CFs [5].

**Figure 8:**
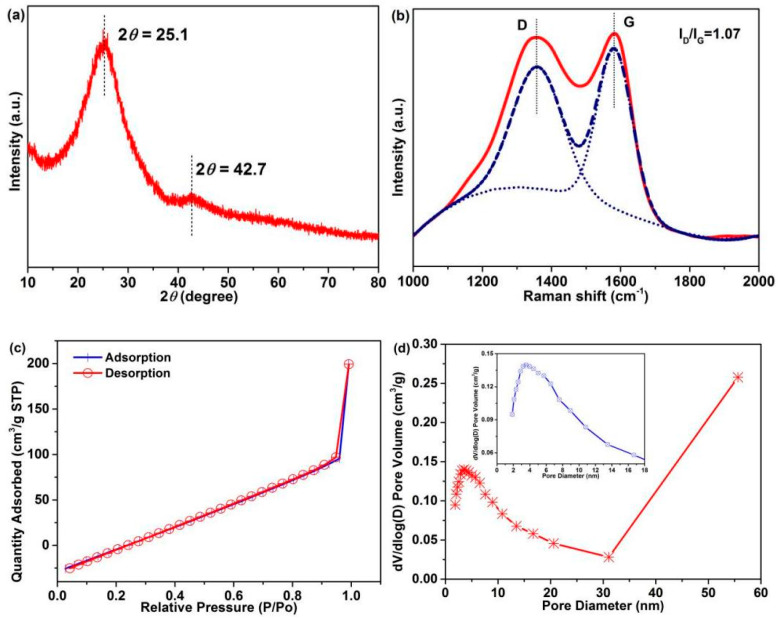
(**a**) XRD pattern, (**b**) Raman spectra, (**c**) nitrogen adsorption–desorption isotherm (**d**) and pore size distributions of the CFs (The inset is part area of pore diameter with 0–18 nm).

The CF crystal structure was confirmed by XRD and Raman characterization, and the graphitization degree was analyzed, which also is a crucial factor for electrochemical performance [5]. Two diffraction peaks at 2*θ* = 25.1° and 42.7°, corresponding to the (002) and (100) crystallographic planes, respectively, are observed in the XRD pattern of CFs (Figure 8a). The broad diffraction peak indicates the disordered carbonaceous structure of CFs [50,51]. According to Scherrer formula [39]:

(1) $$D\;=\;\frac{k\lambda }{\beta (\theta )\cos(\theta )}$$

where *D*, *K*, *β*, *θ*, and *λ* are the average crystallite size, constant, peak width of the physical broadening of sample, Bragg angle of the diffraction peak, and wavelength of the diffraction line, respectively. The average interplanar spacing of the (002) crystal of CFs is calculated to be 0.335 nm, which is close to that of graphite [5]. Figure 8b shows the Raman spectra: Two divided peaks are observed at ~1359.5 cm^−1^ (D-peak) and 1582.7 cm^−1^ (G-peak), corresponding to the hybridized vibration mode of the graphitic plane with disorder or defect structures and the in-plane stretching of sp^2^ in a perfect graphitic structure, respectively. The strength ratio (I_D_/I_G_) of the D-band and G-band represents the graphitization degree of CFs, which is calculated to be 1.07. These results indicate a disordered carbonaceous structure and high graphitization degree of CFs, which render good electrochemical properties. 

The CF pore structure is closely related to the electrochemical performance [52]. The nitrogen adsorption–desorption isotherm (Figure 8c) and pore size distribution (Figure 8d) of CFs were analyzed. As can be observed in Figure 8c, a broken line-type adsorption–desorption isotherm is observed, and the inflection point is the turning point of monolayer adsorption and multilayer adsorption. A sudden increase in the adsorption amount is observed in the high-pressure region of P/P_0_ (0.9–1.0 nm) without almost hysteresis, revealing the presence of macroporous (>50 nm) structures in CFs. The abundant mesoporous (2–50 nm) and macroporous structures in CFs (Figure 8d) are conducive to the penetration of electrolyte, which can generate good carrier transport. Table S3 summarizes the corresponding test results: The surface area of CFs is 4.3 m^2^/g, which is greater than that of PAN fibers (0.1–1.5 m^2^/g).

**Figure 9:**
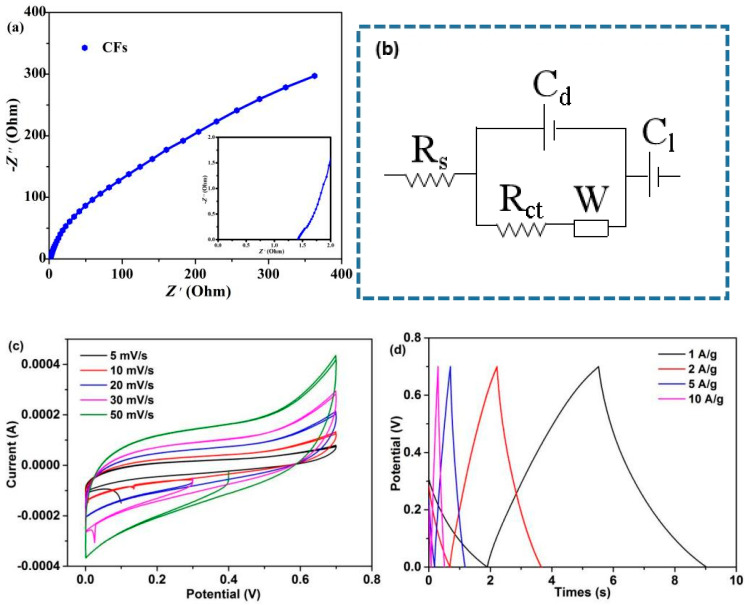
Supercapacitor device: (**a**) Nyquist plots, (**b**) equivalent circuit model, (**c**) CV curves at varies scan rates, and (**d**) GCD curves at varies current densities.

The EIS test of CFs was carried out in the frequency range of 10^−2^–105 Hz. Figure 9a,b and Table S4 show the corresponding Nyquist plots, equivalent circuit model, and fitting parameters, respectively. The Nyquist plot is composed of a semicircle in the high frequency region and a straight line in the low frequency region (Figure 9a). As shown in Figure 9b, R_s_ is internal resistance (including electrolyte ion resistance, active material inherent resistance, and contact resistance between active material and collector), corresponding to the intercept between semicircle and real axis in the high frequency region. R_ct_ is charge transfer resistance, corresponding to the semicircle size in the high frequency region. W is Warbury impedance, C_d_ and C_l_ are electric double layer capacitance and Faraday capacitance, respectively [53]. The simulation results show that the Rs and R_ct_ values of CFs are 1.18 Ω and 0.14 Ω, respectively. The CV curves at 5–50 mV/s scan rates were investigated. The CV curves close to rectangular and symmetrical shapes reveal that the device exhibits an electric double-layer capacitive behavior (Figure 9c) [54], which is consistent with the EIS results. With the increase in the scan rates from 5 to 50 mV/s, the CV curves did not deviate seriously from the rectangular shape, revealing good ionic diffusion [55]. Finally, the GCD test was performed at a current density of 1–10 A/g. As can be observed in Figure 9d, the GCD curve of CFs is close to a symmetrical triangle, indicating that it is close to the ideal electric double-layer capacitive behavior [56]; this result is also demonstrated by EIS and CV curves. The results show that the produced CFs possibly demonstrate good application prospects in electrochemical devices. In recent years, lignin/cellulose-containing blends has gained increasing awareness as a convenient and inexpensive approach to produce CFs with good properties. Research advances have successfully demonstrated that some lignocellulose-based CFs have great application prospects, such as sporting goods and electrodes (Table S5). References [57,58,59,60] are cited in the supplementary materials.

## 4. Conclusions

Inspired by muscles, a cost-effective carbon fiber derived from lignocellulose-based precursors was prepared. IPDI was used as the crosslinking agent, and biomimetic covalent bonds formed by the urethane reaction were used as crosslinking networks to effectively connect poplar lignin and MCC. The spun sub-micro precursor fiber exhibited excellent thermal stability and morphology and high flexibility, rendering an independent filament shape, a uniform diameter, and a high graphitization degree to the derived carbon fiber, as well as good electrochemical performance. These advantages make these fiber products attractive for use in a wide range of applications, including flexible carriers, electrodes, textiles, and sensor devices. Imitating biological muscle tissues to design and develop advanced materials will be a novel and meaningful direction.

## Data Availability

Not applicable.

## Supplementary Materials

The following supporting information can be downloaded at: https://www.mdpi.com/article/10.3390/ma15228068/s1, Figure S1. TG and DTG curves of poplar lignin. Figure S2. Element analysis of lignocellulose-based PFs. Table S1. Amounts of linking modes and monomer for poplar lignin and S/G ratio. Table S2. Elemental compositions of the samples. Table S3. BET results of CFs. Table S4. Fitting values for the parameters of the Nyquist plots. Table S5. Production of CFs from some lignocellulose-based blends and their advantages of characteristic. References [57,58,59,60] are cited in the supplementary materials.
