# Supplementary material for: Inspired by Skeletal Muscles: Study of the Physical and Electrochemical Properties of Derived Lignocellulose-Based Carbon Fibers
Source: Materials (Basel). 2022 Nov 15;15(22):8068. doi: 10.3390/ma15228068 (PMC9694132; doi:10.3390/ma15228068)
Supplement: Supplementary file 1 [file materials-15-08068-s001.zip › materials-1998829-supplementary.pdf]

Supplementary Information

Table S1. Amounts of linking modes and monomer for poplar lignin and S/G ratio.

Sample	*β*–O–4	*β*–*β*	*β*–5	S/G
Poplar lignin	56.57	6.45	2.07	1.54

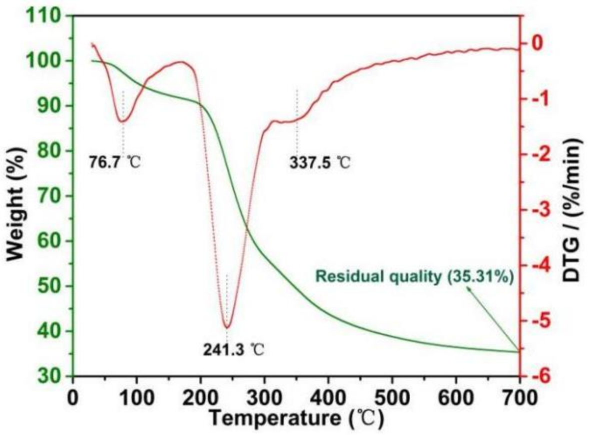

Figure S1. TG and DTG curves of poplar lignin.

Table S2. Elemental compositions of the samples.

Atomic (%)	C	O	N	Sn
Poplar lignin	63.68	35.31	1	_
MCC	56.77	42.65	0.58	_
L-precursor	70.75	22.89	6.22	0.14

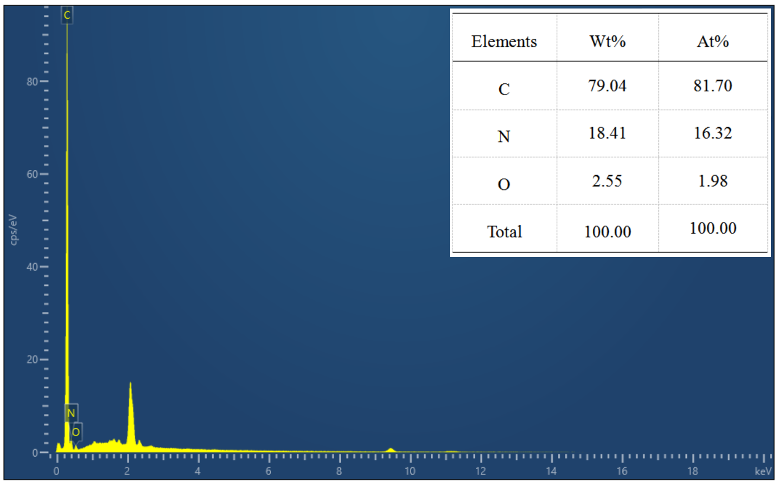

Figure S2. Element analysis of lignocellulose-based PFs.

Table S3. BET results of CFs.

Sample	BET surface area (m^2/g)	Pore volume (cm^3/g)	Pore size (nm)
CFs	4.3489	0.3081	10.0258

Table S4. Fitting values for the parameters of the Nyquist plots.

R_s/Ω	R_{cd}/Ω	W	C_d	C_l
1.18	0.14	0.0059	0.0008	0.0059

Table S5. Production of CFs from some lignocellulose-based blends and their advantages of characteristic.

Carbon fiber precursor	Spinning process	The advantage of characteristic	Refs
Lignin–cellulose acetate	Electrospinning	Derived CFs showed independent filamentous morphology, uniform diameter, large surface area, excellent mechanical property, and power storage capacity	[5]
Eucalyptus dissolving pulp and organosolv/kraft lignin blends	Dry-jet wet spinning	Lignin reduced the vapour adsorption in the fibre and had the positive effect on the carbonisation yield	[57]
Plant protein–lignin	Electrospinning	The well-engineered structural characteristics	[58]
Cellulose–chitosan	Dry-jet wet spinning	Improved the carbon yield and structural properties	[59]
Lignin/PAN	Electrospinning	Derived CFs had good spinnability, high crystallization and mechanical strength	[29]
Cotton and cellulose acetate blend with PAN	Electrospinning	Low cost and environmentally friendly alternatives to conventional CF precursors and processing solvents	[60]
Kraft lignin blended with kraft pulps	Dry-jet wet-spinning	The best CFs are stronger and stiffer than those produced by melt-spinning of softwood-based lignins alone	[18]
Extracted poplar lignin–MCC/PAN	Electrospinning	Nature-inspired biomimetic structural characteristics and derived CFs had excellent morphology, high graphitization degree and good electrochemical properties	—
